# Personalized and Culturally Tailored Features of Mobile Apps for Gestational Diabetes Mellitus and Their Impact on Patient Self-Management: Scoping Review

**DOI:** 10.2196/58327

**Published:** 2024-12-12

**Authors:** Catherine Jones, Yi Cui, Ruth Jeminiwa, Elina Bajracharya, Katie Chang, Tony Ma

**Affiliations:** 1 Werbie LLC, 4803 Dover Court, Bethesda, MD, 20816, United States, 1 301 283 8703; 2 Benten Technologies, Manassas, VA, United States; 3 Thomas Jefferson University, Philadelphia, PA, United States

**Keywords:** gestational diabetes, pregnancy, cultural tailoring, cultural adaptation, personalization, maternal health, mobile health, mHealth, smartphone, mobile app, mobile phone, self management; social determinants of health

## Abstract

**Background:**

Gestational diabetes mellitus (GDM) is an increasingly common high-risk pregnancy condition requiring intensive daily self-management, placing the burden of care directly on the patient. Understanding personal and cultural differences among patients is critical for delivering optimal support for GDM self-management, particularly in high-risk populations. Although mobile apps for GDM self-management are being used, limited research has been done on the personalized and culturally tailored features of these apps and their impact on patient self-management.

**Objective:**

This scoping review aims to explore the extent to which published studies report the integration and effectiveness of personalized and culturally tailored features in GDM mobile apps for patient self-management support.

**Methods:**

We examined English-language peer-reviewed articles published between October 2016 and May 2023 from PubMed, CINAHL, PsycINFO, ClinicalTrials.gov, Proquest Research Library, and Google Scholar using search terms related to digital tools, diabetes, pregnancy, and cultural tailoring. We reviewed eligible articles and extracted data using the Arskey and O’Malley methodological framework.

**Results:**

Our search yielded a total of 1772 articles after the removal of duplicates and 158 articles for full-text review. A total of 21 articles that researched 15 GDM mobile apps were selected for data extraction. Our results demonstrated the stark contrast between the number of GDM mobile apps with personalized features for the individual user (all 15 mobile apps) and those culturally tailored for a specific population (only 3 of the 15 mobile apps). Our findings showed that GDM mobile apps with personalized and culturally tailored features were perceived to be useful to patients and had the potential to improve patients’ adherence to glycemic control and nutrition plans.

**Conclusions:**

There is a strong need for increased research and development to foster the implementation of personalized and culturally tailored features in GDM mobile apps for self-management that cater to patients from diverse backgrounds and ethnicities. Personalized and culturally tailored features have the potential to serve the unique needs of patients more efficiently and effectively than generic features alone; however, the impacts of such features still need to be adequately studied. Recommendations for future research include examining the cultural needs of different ethnicities within the increasingly diverse US population in the context of GDM self-management, conducting participatory-based research with these groups, and designing human-centered mobile health solutions for both patients and providers.

## Introduction

Gestational diabetes mellitus (GDM) is an increasingly common high-risk condition in pregnancy that creates potential short- and long-term complications for the mother-child dyad [1,2]. It is defined as any degree of glucose intolerance that begins or is first recognized during pregnancy and resolves postpartum [3]. GDM confers maternal and fetal risks related to the degree of hyperglycemia as well as chronic complications and comorbidities of the patient [4]. Specific risks to women with prepregnancy diabetes and diabetes developed in pregnancy include preeclampsia, preterm delivery, fetal anomalies (ie, intrauterine growth restriction, small for gestational age, macrosomia, and morphologic anomalies), neonatal hypoglycemia, hyperbilirubinemia, and neonatal respiratory distress syndrome, among others [5,6]. In addition, diabetes in pregnancy may increase the risk of obesity [7], hypertension, and type 2 diabetes in the mother and her offspring later in life [8-10].

Although there is an increasing number of women entering pregnancy with preexisting diabetes, paralleling the global obesity epidemic, there is also concurrently a dramatic increase in the reported rates of GDM [6]. In the United States, ethnic minority women (including Native American, Asian, Hispanic, Latino, and Black women) and women of lower socioeconomic status are often disproportionately affected by both preexisting diabetes and GDM [11-13]. Rates of GDM among minorities have increased in the past 20 years by 10%-100%, making minorities twice as likely as White women to develop GDM [14].

Further, ethnic minority groups in the United States, which are expected to increase from 41.8% of the population in 2019 to 49.3% by 2045 [15], often have higher rates of diabetes-associated morbidity and mortality [16]. Racial and ethnic differences in GDM prevalence and GDM-related adverse perinatal outcomes are well-documented [17]. Even though the prevalence of GDM is higher among Hispanic and Asian women, non-Hispanic Black women have the highest rate of GDM-related adverse outcomes, including preeclampsia, preterm delivery, and neonatal hypoglycemia [18]. Substantial racial disparities in the emergence of type 2 diabetes after GDM show a 4-fold increased risk among Black individuals and 3-fold increased risk among Hispanic and South and Southeast Asian individuals relative to White individuals [19]. Although non-Hispanic Black women have a lower risk of developing GDM during pregnancy, they are 10 times more likely to develop type 2 diabetes following birth [20].

After a GDM diagnosis, a patient is responsible for carrying out complex, daily self-management tasks with close monitoring by a multidisciplinary care team focused on maintaining optimal glycemic control to avoid hyperglycemia, carbohydrate intolerance, and other complications [21]. With proper care and support, approximately 75%-90% of GDM cases can be managed with lifestyle adjustments alone, avoiding the need for pharmacological interventions in the form of medications and insulin [22]. Women from racial and ethnic minorities and vulnerable populations, who already experience greater rates of preterm birth, stillbirth, and maternal mortality, often face additional barriers to GDM self-management, such as language, health literacy, lack of educational materials, and inability to meet the requirements of their nutrition plans [23].

The use of mobile apps for GDM self-management is gaining traction in the United States and globally, with new software entering the market at an increased rate. Despite this, addressing the unique needs of diverse GDM populations is not receiving enough attention. With the rapid growth of mobile health (mHealth) technologies, there is an opportunity to enhance self-management strategies for these women. Features for mHealth, such as personalization and cultural tailoring, are needed to create sustainable behavior changes necessary for successful GDM self-management.

Research that defines personalization and cultural tailoring as 2 distinct concepts and explores their impacts on GDM self-management will help refine and optimize intervention models [24]. Personalization caters to users on an individual level, not at the group, community, or population level [25]. In the context of GDM mobile app design, personalization can be broadly defined as the inclusion of features that customize a user’s experience based on health needs, engagement preferences, accepted forms of feedback and communication, and other individualized features (eg, goal setting and appointment reminders). Patient data collected by the app informs treatment plans related to evolving health status (ie, maternal age, gestational age, maternal medical history, comorbidities, medications, and lifestyle) and can be personalized to enhance the user’s self-management and the care team’s ability to provide feedback on key metrics including blood glucose levels, nutrition plans, and physical activity.

Cultural tailoring (sometimes called cultural adaptation) refers to the adaptation of the intervention design, materials, and other components to reflect the cultural needs and preferences of the priority population. This process requires an understanding of the population’s values, attitudes, history, and other influences on behavior. Cultural tailoring is intended to maximize rates of participant recruitment for studies, increase rates of completion or adherence, enhance accuracy in language and understanding in communications, and increase positive outcomes from treatment [26].

Barerra et al [27] identified 2 levels of cultural adaptations: surface structure adaptation and deep structure adaption. Surface structure features include: bilingual and bicultural materials, language translation or back-translation of materials, inclusion of ethnic lifestyle features (eg, food and music), use of community health workers to incorporate traditional healing practices (eg, acupuncture), incorporation of culturally familiar formats and activities (eg, social support structures), and inclusion of same race or ethnicity role models. Deep structure adaptation refers to the incorporation of 1 or more components of cultural values in intervention design or implementation, involvement of the family and possibly peers in the intervention and decision making, adjustment of material to literacy levels of the participants, and use of social support and networks [27]. Culturally adapted interventions have been shown to be effective in promoting health education, healthy eating, and physical activity, specifically among ethnic minorities and underserved populations [28,29].

To the best of our knowledge, this is the first review to examine personalization and cultural tailoring in GDM mobile apps with the goals of: (1) determining the extent to which GDM mobile apps used in published studies provide personalized and culturally tailored features for self-management support for patients and (2) assessing the impact of these GDM mobile apps with personalized and culturally tailored features on patient self-management.

## Methods

### Study Design

#### Overview

We followed Arksey and O’Malley’s 6-step methodological framework [30] to conduct this scoping review: (1) clarify the research question, (2) identify relevant studies, (3) select relevant studies, (4) chart the data, and (5) collate, summarize, and report results. We did not include the optional sixth step, a consultation exercise with stakeholders, because we did not engage stakeholders in any stages of this review. Reporting techniques were guided by the PRISMA-ScR (Preferred Reporting Items for Systematic Reviews and Meta-Analyses extension for Scoping Reviews) checklist [31].

#### Framework Step 1: Clarifying the Research Question

The two research questions for this scoping review were as follows:

1. Do the GDM mobile apps that were utilized in published studies offer patients personalized and culturally tailored features for GDM self-management?
2. Do the personalized and culturally tailored features have a positive effect on patient self-management?

#### Framework Step 2: Identifying Relevant Studies

To comprehensively identify relevant studies in recent literature, this review conducted search strategies in PubMed, Scopus, CINAHL, PsycINFO, ClinicalTrials.gov, Proquest Research Library, and Google Scholar. English language studies published between October 2016 to May 2023 were eligible for inclusion in the review. We limited our searches to studies published in the last 7 years because software technology evolves at a rapid rate, and we wanted to ensure that the apps were all at a similar level of technological advancement. With Google Scholar, we limited the search to include the top 200 articles ranked by relevance. Our search strategy, developed under the guidance of a research librarian, included “Diabetes, Gestational”[MeSH] OR “Pregnancy in Diabetics”[MeSH] OR “Pregnancy”[MeSH] OR “Postnatal Care”[MeSH] OR “Prenatal Care”[MeSH] OR “Maternal Health Services”[MeSH] OR “Women’s Health”[MeSH] OR “Women’s Health Services”[MeSH] AND “Diabetes Mellitus”[MeSH] OR “Diabetes Insipidus”[MeSH] OR “Diabetes Mellitus, Type 2”[MeSH] OR “Diabetes Mellitus, Type 1”[MeSH] OR “Blood Glucose/metabolism”[MeSH] OR “Blood Glucose Self-Monitoring”[MeSH] OR “Glycemic Control”[MeSH] AND “Mobile Applications”[MeSH] OR “mobile application*”[tw] OR “mobile app”[tw] OR “mobile apps”[tw] OR “mobile technolog*”[tw] OR “mobile healthcare”[tw] OR “mHealth”[tw] OR “Smartphone”[MeSH]) AND (“Culturally Appropriate Technology”[MeSH] OR “Cultural Characteristics”[MeSH] OR “cultural tailoring” OR “cultural factors” OR “cultural barriers” OR “Culture”[MeSH] OR “Cultural diversity”[tw] OR “Cultural deprivation”[tw] OR “Cultural competency”[tw] OR “Cultural characte*”[tw] OR “cultural sensitivity”[tw] OR “cultural concordance”[tw] OR “Multilingualism”[MeSH] OR sociocultural OR “Hispanic or Latino”[MeSH] OR “Black or African American”[MeSH] OR “Asian American Native Hawaiian and Pacific Islander”[MeSH] OR “American Indian or Alaska Native”[MeSH] OR “Attitude to Computers”[MeSH] OR “Health Literacy”[MeSH] OR “Digital literacy”[tw] OR “Health Liter*”[tw] OR “health knowledge”[tw] OR “health understanding”[tw] OR “Health Knowledge, Attitudes, Practice”[MeSH] OR “Patient Education as Topic”[MeSH] OR “Patient Acceptance of Health Care”[MeSH] OR “Treatment Adherence and Compliance”[MeSH] OR “Patient Compliance”[MeSH] OR “methods”[subheading] OR “psychology”[subheading] OR “education”[subheading] OR “Communication”[MeSH].

#### Framework Step 3: Study Selection

We used an iterative process to develop inclusion and exclusion criteria to identify articles and research studies relevant to our research questions. Our inclusion criteria for articles were: (1) the study involved a mobile app, and (2) the mobile app used in the study was explicitly designed for GDM self-management. For example, studies that used mainstream mobile apps (eg, WhatsApp or WeChat) to support GDM self-management were excluded from the study. We excluded studies that: (1) did not provide a detailed description of app features, (2) were not yet published or provided insufficient details about the study (eg, poster presentations, unpublished ClinicalTrials.gov studies), (3) were classified as reviews (eg, scoping reviews, systematic reviews, meta-analysis, and editorials), and (4) were not written in English. Two authors (CJ and YC) reviewed the full text of each article independently. A third reviewer (RJ) reviewed the included articles to ensure they met the eligibility criteria.

#### Framework Step 4: Charting the Data

We created an Excel (Microsoft Corp) file to chart, collate, and summarize the results. We recorded both characteristics of the studies and specific information pertaining to the 2 research questions, including data on author(s), year of publication, country, study design, participants, app name, personalization features, culturally tailored features, and the app’s impact on GDM self-management.

#### Framework Step 5: Collating, Summarizing, and Analyzing the Results

The final process involved collating, summarizing, and analyzing the data and interpreting and reporting the results. The first and second authors independently examined the Excel file for accuracy and to identify common themes. We compared the various features of the mobile apps and the impacts they had on patient self-management in Multimedia Appendix 1. The 15 mobile apps included: Habits-GDM (Singapore) [32,33], SweetMama (United States) [34,35], Mother (Australia) [36], mAPP (Korea) [37], Pregnant+ (Norway) [38,39], myDiabby (France) [40], Dnurse (China) [41], GlucoseBuddy (United States) [42], GDm-Health (United Kingdom) [43], MobiGuide (Spain) [44,45], Stay-Active (United Kingdom) [46], and 4 apps that did not have names, which we refer to as App #1 (Korea) [47], App #2 (Korea) [48], App #3 (Malaysia) [49], and App #4 (New Zealand) [50]. Study characteristics and features of personalization and cultural tailoring in GDM mobile apps incorporated study design information, stated aims, and lists of personalized and culturally tailored features. The data were analyzed for common themes.

## Results

### Overview of the Screening Results

**Figure 1.**
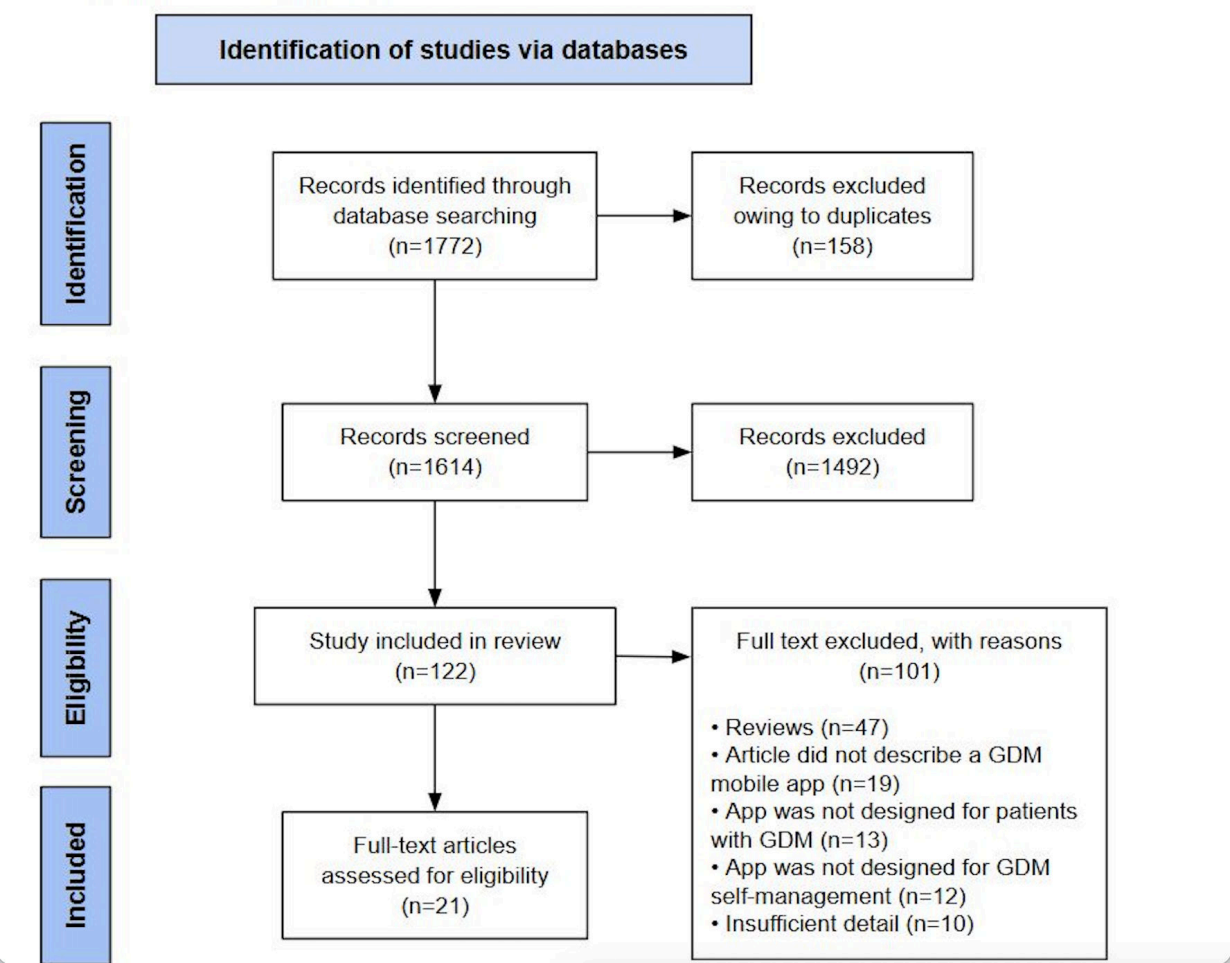
PRISMA flowchart. GDM: gestational diabetes mellitus; PRISMA: Preferred Reporting Items for Systematic Meta-Analysis.

The literature searches yielded 1772 records. After removing duplicates, 1614 records were retained for title and abstract review. A total of 122 full-text studies were assessed for eligibility. After completing the study selection process, 21 studies were included in the review, as demonstrated in Figure 1.

The first research question was to determine if and to what extent mobile apps offer patients personalized and culturally tailored features for GDM self-management. Key themes for personalized app features included: live chat (ie, direct communication with a provider to answer patient questions in real time), messaging (eg, educational tips, motivational messages, and reminders, as well as messages to patients when a blood glucose reading was too high), glycemic control, nutrition support (ie, personalized nutritional advice from care teams based on patient logging data such as blood glucose levels), physical activity, medication support, educational materials, and postpartum care. Our findings showed that all 15 mobile apps had some element of personalization, but only 3 of 15 apps included elements of culturally tailored features: Habits-GDM, SweetMama, and Pregnant+.

The second question examined if these GDM mobile apps with personalized and culturally tailored features had a positive effect on patient self-management. Key themes for culturally tailored mobile app features included educational materials, nutritional support (eg, nutritional information adapted or tailored to align with a specific cultural group, including inclusion of an ethnic-focused food database for logging), language, and health literacy level. The results of the 7 studies using the 3 personalized and culturally tailored GDM mobile apps (Multimedia Appendix 2) showed that personalization and cultural tailoring have the potential to positively impact GDM self-management. When combined with personalization, both culturally tailored educational material and nutrition support showed an impact on increased patient motivation for self-management of blood glucose values, while culturally tailored nutrition support showed a positive impact on improved glycemic control and neonatal outcomes, as well as increased patient motivation and confidence in self-managing GDM. Adjusted health literacy levels showed increased utilization and ease of use, improved user satisfaction, and greater perceived usefulness. The effects of language tailoring were not specifically studied in the articles; therefore, the authors could not draw conclusions about this factor.

### Extent of Personalized GDM Mobile App Features

All 15 GDM mobile apps in this scoping review had personalized features, which were grouped in 8 categories. Our results showed that 4 apps offered live chat and all 15 offered patient SMS text messaging on some level. Under the category of glycemic control, all the apps except SweetMama and Stay-Active offered feedback on patient blood glucose logging. In addition, 12 apps offered personalized nutrition support, such as dietary information based on eating preferences, meal logging, and feedback on logging in the form of in-app charts. Of the 15 apps, 11 offered support or tips for physical activity, and 7 offered medication support such as reminders or assistance with dosing insulin. All the apps except MyDiabby, MobiGuide, App #3, and App #4 offered personalized educational materials, such as the ability to choose GDM pregnancy or postpartum messages and what time of day to receive them. There were 4 apps that offered postpartum care tips (eg, breastfeeding encouragement and postpartum weight loss assistance) and encouraged the 6-week postpartum oral glucose tolerance test (OGTT). Table 1 illustrates the 8 categories of personalized mobile app features studied in this review.

**Table 1.** Personalized features in mobile apps for GDM.^a^

Mobile app	Personalized mobile app features							
	Live chat	SMS text messages	Glycemic control	Nutrition support	Physical activity	Medication support	Education materials	Postpartum care
Habits-GDM		√	√	√	√		√	
SweetMama		√					√	
Pregnant+		√	√	√	√		√	√
Mother	√	√	√	√	√		√	
mAPP		√	√	√	√	√	√	√
myDiabby		√	√	√		√		
Dnurse	√	√	√	√	√	√	√	
GlucoseBuddy	√	√	√	√	√	√	√	√
GDm-Health		√	√	√	√	√	√	
MobiGuide		√	√					
Stay-Active	√	√			√		√	
App #1 [47]		√	√	√	√	√	√	√
App #2 [48]		√	√	√	√		√	
App #3 [49]		√	√	√				
App #4 [50]		√	√	√	√	√		

a GDM: gestational diabetes mellitus.

### Extent of Culturally Tailored GDM Mobile App Features

Examining the 3 GDM mobile apps with culturally tailored features showed that 2 apps (Pregnant+ and SweetMama) offered culturally tailored educational materials (ie, self-care educational guidance) and all 3 apps (Pregnant+, SweetMama, and Habits-GDM) offered culturally tailored nutrition support (ie, dietary treatment of GDM for optimal glycemic control). Cultural tailoring by language (patient’s ability to select a preferred language) and health literacy level (the degree to which individuals can find, understand, and use information and services to inform health-related decisions and actions for themselves and others) was only available in SweetMama. These results are laid out in Table 2.

**Table 2.** Mobile apps for GDM that include culturally tailored educational materials, nutrition support, language, and health literacy levels.^a^

Mobile app	Culturally tailored mobile app features with surface and deep adaptations			
	Culturally tailored educational materials (surface)	Culturally tailored nutrition support (surface)	Culturally tailored language (surface)	Culturally tailored health literacy levels (deep)
Pregnant+	√	√	√	
SweetMama	√	√		√
Habits-GDM		√		

a GDM: gestational diabetes mellitus.

### Extent of Surface and Deep Structure Adaptations of Culturally Tailored Features

According to Barerra’s adaptation model, the 3 categories of culturally tailored app featured in this review can all be classified as surface level. They included bilingual materials (Pregnant+); ethnic lifestyle features, such as nutrition support (Pregnant+, SweetMama, and Habits-GDM); and culturally familiar formats in educational materials (Pregnant+ and SweetMama). The only app that included deep structure adaptation including cultural health literacy levels was SweetMama.

### Impacts of Culturally Tailored Educational Materials and Personalization on Self-Management

#### Pregnant+

Culturally tailored educational materials in this mobile app designed in Norway included information on GDM, physical activity, nutrition, and breastfeeding in 3 different languages: Norwegian, Urdu, and Somali. The physical activity information in Pregnant+ included the benefits of being physically active and examples of healthy activities. Nutrition support included materials on ethnic foods, while general educational materials discussed the importance of check-ups during pregnancy and postpartum, as well as the advantages of breastfeeding. The personalized features in Pregnant+ included messaging in the form of personalized feedback on glycemic control (users received a green smiley face indicating a normal blood glucose value and a red smiley face indicating a high value) and physical activity (users recorded personal goals related to active living), as well as educational materials tailored to pregnancy and the postpartum period. Skar et al [51] conducted a qualitative analysis using semistructured interviews with 17 participants from a previous Pregnant+ randomized control study (RCT) to assess the app users’ experience. Results from these interviews showed that Pregnant+ was useful to patients for controlling blood glucose levels and increasing confidence and motivation in overall GDM self-management.

#### SweetMama

Culturally tailored educational materials in this mobile app designed in the United States included educational resources and SMS text messaging suitable for various health literacy levels. Personalized features included bidirectional messaging with providers through an in-app messaging center, individualized goals, and educational materials tailored to gestational age that were designed to promote health knowledge. Yee et al [35] conducted a study using focus groups with patients and providers to solicit feedback on the app. A total of 16 low-income patients diagnosed with GDM or type 2 diabetes and 29 health care providers with experience treating or educating patients with GDM participated. The qualitative assessment revealed that both patients and providers were satisfied with the information provided in SweetMama. Specifically, participants expressed that the culturally tailored content was easy to understand, practical, and helpful. Content delivery in the form of short text messages (promoting one tip at a time) was seen by patients as an effective way to present information.

### Impacts of Culturally Tailored Nutrition Support and Personalization on Self-Management

#### Pregnant+

Culturally tailored nutrition support in this Norwegian app included information on healthy diets and recommendations for healthy drinks. A link to the Norwegian Diabetes Foundation was provided to give women access to recipes and information related to a GDM pregnancy. A Healthy Diet Score (HDS-P+) was used to assess healthy eating habits based on dietary logging. Borgen et al [39] conducted an RCT with 238 patients to examine the effect of Pregnant+ on the 2-hour postpartum OGTT. All participants in the study received the standard care and the intervention group was given access to Pregnant+. Primary analysis showed no significant difference in 2-hour OGTT at 3 months postpartum between the intervention and control groups (6.7 mmol/L vs 6.0 mmol/L, *P*=.54). Secondary analysis of HDS-P+ scores showed an overall improvement in patients’ healthy eating behaviors from 40.4 at baseline to 56.1 for the control group and 55.34 for the intervention group at the 36-week follow-up. However, no significant difference in average HDS-P+ scores (55.3 vs 56.1, *P*=.65) was observed. Additionally, there were variations in how patients perceived the culturally tailored dietary information. Some felt it was easy to follow, while others felt it was difficult to maintain decreased amounts of carbohydrates, particularly related to portion sizes for rice and pasta.

#### Habits-GDM

Culturally tailored nutrition support in the Habits-GDM app, designed in Singapore, included an ethnic food database adapted to incorporate common Singaporean food derived from Chinese, Malay, Indian, and Western cultures. It also provided 12 “bite-sized” interactive lessons on nutrition and physical activity, as well as comprehensive tracking tools for self-monitoring of blood glucose levels, diet, physical activity, and weight gain. The personalized features of this mobile app included messaging in the form of personalized feedback on glycemic control (automatic messages in response to high blood glucose readings), nutrition support (meal logging), physical activity (tracking the number of daily steps taken using the participants’ built-in phone pedometers), and a messaging platform that allowed users to ask questions, schedule appointments, and request blood glucose testing strips from health care professionals. Yew et al [32] conducted an RCT with 340 patients with GDM to evaluate Habits-GDM’s efficacy in reducing excessive gestational weight gain. Although no significant difference was observed between participants using the app plus usual care versus those receiving usual care alone, secondary analysis revealed that Habits-GDM led to improvements in glycemic control and neonatal outcomes. Participants using Habits-GDM plus usual care achieved significantly lower average blood glucose readings (mean 5.40, SD 0.53 vs mean 5.54, SD 0.53, *P*=.01) and experienced fewer overall neonatal complications (38.1% vs 53.7%, *P*=.006) when compared to those receiving usual care alone.

In another study, Surendran et al [33] conducted a quantitative analysis of usage data and semistructured interviews with 14 participants from the Habits-GDM RCT by Yew et al [32]. Results showed that diet tracking was the least used app feature, with participants logging only an average of 0.88 meals per week. A majority of participants (12/14) reported negative experiences with diet tracking due to issues with the food database around the limited representation of ethnic food items. Other personalized app features, including the messaging platform, were perceived as useful for GDM self-management by the participants.

#### SweetMama

Results from a focus group by Yee et al [35] showed that both patients and providers (6/16 patients; 7/29 providers) desired more information on nutrition education and support, including the glycemic index of food items, dietary recommendations for patients, and recipes. Based on these findings, a revised version of the mobile app was created with a library component that included a recipe repository and educational materials from culturally tailored diabetes-specific sources (eg, the American Diabetes Association). The study did not collect any feedback on the SweetMama app with the revised nutrition features.

### Impact of Culturally Tailored Language on Self-Management (Pregnant+)

This was the only mobile app that offered culturally tailored language options for users: Norwegian, Urdu, and Somali. The study’s authors did not investigate the impact of culturally tailored language on GDM self-management in any of the 3 studies conducted using this app; therefore, the impact of language could not be determined.

### Impact of Culturally Tailored Health Literacy Levels and Personalization on Self-Management (SweetMama)

The study participants included 22 low-income pregnant women with GDM or type 2 diabetes. Steinberg et al [34] analyzed app data from a 2-week usability assessment to examine user health and behavioral characteristics including diabetes type, electronic health literacy (computer literacy related to health information processing), general health literacy (ability to obtain and process health information), patient activation (patient engagement in the health care process), and diabetes self-efficacy (psychosocial self-efficacy of individuals with diabetes using the Diabetes Empowerment Scale–Short Form). Results revealed participants with GDM had higher average minutes of use per session (mean 4.0, SD 2.9 minutes vs mean 2.5, SD 0.9 minutes) and total duration of use (mean 28.1, SD 17.0 minutes vs mean 18.2, SD 12.3 minutes) compared to pregnant patients with type 2 diabetes. Participants with greater electronic health literacy, lower patient activation, and higher diabetes self-efficacy also reported greater total duration of use. Study participants expressed that the personalized messaging with content based on gestational age was practical and helpful. Additionally, content delivered in the form of short text messages (one tip at a time) was seen by users as an effective way to present information, improve user satisfaction, and enhance the perceived usefulness of the information received.

## Discussion

### Impacts of Personalized and Cultural Tailoring Features of GDM Mobile Apps

The use of mobile apps to assist and empower patients to self-manage GDM has shown potential benefits over the traditional model of care that is still in use today, such as logging with paper and pen and communication with doctors and care teams primarily during in-person prenatal and postpartum visits. Despite advances in mobile app technology, patients continue to struggle with daily GDM self-management protocols due to cultural and language barriers, inappropriate health literacy levels, and lack of access to health care providers or the evidence-based interventions they need. One important strategy to overcome some of these hurdles is to personalize and culturally tailor mobile apps for GDM to serve specific racial and ethnic minority groups and vulnerable populations.

This scoping review highlights how important it is to consider individual patient needs as well as the broader context and nuances of the patient’s social and cultural environment. Results demonstrated that personalized app features were far more common than culturally tailored features and both types of features were useful in self-managing blood glucose levels, thereby increasing confidence, motivation, and understanding.

### Challenges of Culturally Tailoring Features in GDM Mobile Apps

Ultimately, the goal of GDM mobile apps should be to facilitate medical care teams, family, community, and peers to empower patients to produce positive outcomes. The use of cultural tailoring has the ability to leverage cultural strengths by fostering a sense of belonging and community as a means to overcome cultural barriers, stigmas, health disparities, and inequities. Recognizing and respecting cultural identities, languages, ethnic foods, values, and customs, combined with providing culturally appropriate health education, results in patients more likely to feel supported, understood, and motivated, which can lead to increased adoption and sustainability of healthy behaviors during pregnancy and the postpartum period.

Addressing cultural tailoring in the research and development of GDM mobile apps requires raising cultural awareness and knowledge, providing training and education to both health care professionals and IT developers, and allocating the financial and human resources needed to cover additional costs, such as hiring specialists on research teams. Other factors include ensuring diversity among the health care providers who are administering the mHealth intervention and using evidence-based and applicable guidelines to get feedback and tweak features as needed. With these requirements often being a challenge to meet, or completely unattainable, mHealth interventions and programs for self-managing GDM understandably rely on standardized, nontailored approaches and universal guidelines, which do not account for patient diversity, often leading to overlooked or misunderstood needs.

### Implications for Future Research and Development

As this review demonstrates, research to effectively measure the impact of cultural tailoring in mHealth is tricky. Joo et al [15] pointed out areas of difficulty researchers might encounter when quantifying the impact of cultural tailoring, including unclear guidelines for cultural tailoring in general; intervention implementation (eg, inadequate training staff, language barriers, cultural competency barriers); low attention and retention rates of participants in research settings; defining measures (eg, process, dosage, fidelity); and defining how to measure the evaluation of care in comparison to standard care [26]. Looking at the study designs of the culturally tailored apps in this review, only 1, Habits-GDM, specifically mentioned cultural tailoring in its study aims.

To advance the understanding of patient engagement and efficacy, future research should include components of Barrera’s surface structure and deep structure adaptations developed in iterative stages along with metrics to determine the effectiveness of these adaptations relative to their original versions. A close collaboration should be established between researchers and developers using a human-centered design model, defined as a sociotechnical approach to innovation that considers human activities in a social environment.

Multifaceted social and structural influences, which can intricately shape the course of GDM and patient health outcomes, should also be addressed. In the United States, these multidimensional influences are commonly referred to as social determinants of health, encompassing access to housing, food, education, work, and health care. Structural determinants of health (eg, policies, economic systems, and social hierarchies, such as racism) invariably impact social determinants of health, creating health disparities that are particularly pronounced among racial and ethnic minority groups. Adequately addressing these upstream determinants of health can directly impact personal agency downstream, including the ability to self-manage GDM.

A study by Gonzales et al [52] concluded that co-design with intended users, even small sample sizes of minority populations (often described as participatory-based research), could address and find solutions for challenges, such as barriers relating to digital and health literacy, language, nutrition, education, adherence, trust, and internet access and devices. Inability to access the internet is commonly referred to as the racial digital divide in the United States, which persists despite some advances in access to wireless infrastructure. According to a 2021 Brookings Institution report, 15%-24% of American lack any sort of broadband connection to the internet, limiting their ability to use mHealth technology. This digital divide increases with income disparities in both rural and urban areas [53].

Using an established co-design framework, such as the PRECEDE-PROCEED model, can effectively guide the participatory process of intervention design [54]. This involves conducting formative research, such as focus group discussions and in-depth interviews, with stakeholders throughout the design process and focusing on the constructs of predisposing (eg, health literacy, access to the internet), reinforcing (eg, motivational messages and rewards), and enabling (eg, access to evidence-based content) factors that play a critical role in influencing behavior change among target users. Building iteratively based on feedback from patients and their providers, and any other stakeholders, can improve feasibility and usability, digital literacy, and trust among users. In this review, 2 app studies, Pregnant+ and SweetMama, specifically focused on using an iterative process of patient and provider feedback to dictate design. The bottom line is that even if a mobile app provides high-quality, evidence-based content, the value of an intervention is limited if the information does not adequately match and address the usability, accessibility, readability, engagement, and health literacy needs of the target audience [55].

### Strengths and Limitations

The main strength of this scoping study lies in its ability to clearly and comprehensively map out and analyze the field of research on mobile apps for GDM self-management, particularly their inclusion of personalized and culturally tailored app features. Given that cultural tailoring is not widely incorporated into GDM mobile apps, this work has the potential to significantly inform future research and development in this field. By presenting a clear distinction between personalization and cultural tailoring, this study provides a valuable methodological review that can guide future research of mobile apps.

There were numerous limitations to this study. The study’s inclusion of only English-language articles may have overlooked GDM research in other languages; however, the review did include mobile apps from 11 countries. There may also be GDM mobile apps in existence that are culturally tailored but that have yet to be used in formal research, or the research may not be published, or the app may currently be in the development process. Although we strove to capture as many articles as possible, we limited ourselves to 5 databases and looked at a 5-year period starting from October 2016 to May 2023 to ensure that the technology being used was not outdated. This may have caused some articles on this topic to be omitted. Recruitment of minorities, ethnic communities, or marginalized groups has been known to be difficult in research, which may have limited the power to detect statistically significant results in certain studies.

The descriptive nature of this review precludes any conclusions about causality, while the absence of a meta-analysis means that findings were not quantitatively synthesized, limiting the ability to generalize results. Future research should address these limitations by incorporating a more detailed analysis and considering the inclusion of non–English language studies and gray literature. Finally, this scoping study concentrated solely on GDM self-management related to the use of mobile apps, a narrow scope that excluded other social and structural influences that can impact a patient’s health and health care.

### Conclusions

There is a strong need for increased research and development to foster the implementation of personalized and culturally tailored features in GDM mobile apps for self-management designed for patients from diverse backgrounds and ethnicities. Personalized and culturally tailored features have the potential to serve the unique needs of patients more efficiently and effectively than generic features alone. However, their impacts have not been adequately studied. Recommendations for future research include studying the cultural needs of different ethnicities in the context of GDM self-management (particularly in the increasingly diverse US population), conducting participatory-based research with these groups, and designing human-centered mobile app solutions to serve both patients and providers.

## Supplementary material

10.2196/58327 Multimedia Appendix 1 Study characteristics and features of cultural tailoring and personalization in gestational diabetes mellitus mobile app.

10.2196/58327 Multimedia Appendix 2 Presentation of 3 mobile apps for gestational diabetes mellitus used in 7 studies including app name, study and study design, personalized and culturally tailored features, and the impact of gestational diabetes mellitus self-management.

10.2196/58327 Checklist 1 PRISMA-ScR checklist.

## Abbreviations

GDM: gestational diabetes mellitus
HDS-P+: Healthy Diet Score
mHealth: mobile health
OGTT: oral glucose tolerance test
PRISMA-ScR: Preferred Reporting Items for Systematic Reviews and Meta-Analyses extension for Scoping Reviews
RCT: randomized controlled trial
