# Supplementary material for: Personalized and Culturally Tailored Features of Mobile Apps for Gestational Diabetes Mellitus and Their Impact on Patient Self-Management: Scoping Review
Source: JMIR Diabetes. 2024 Dec 12;9:e58327. doi: 10.2196/58327 (PMC11683422; doi:10.2196/58327)
Supplement: Multimedia Appendix 1 [file diabetes-v9-e58327-s001.docx]

**Multimedia Appendix 1. Study characteristics and features of cultural tailoring and personalization in GDM mobile app.**

| Study (first author, and date) | Study Design, Participants, Country | App Name | Stated Aim(s) of Study | Culturally Tailored Features | Personalized Features |
| --- | --- | --- | --- | --- | --- |
| Yew, 2021 | RCTa, n = 340 GDMb patients, Singapore | Habits-GDM | To examine whether the coaching program can prevent excessive gestational weight gain and improve glycemic control and maternal and neonatal outcomes | Nutrition support  Educational materials  Customized database of Chinese, Malay, and Indian foods for logging | Messaging, glycemic control, nutrition support, and PAc  Chat function with HCPsd to answer patient questions within 24 hours; when a BGLe was high patients were cued through automated messages to record their diet in the preceding 2 to 4 hours |
| Surendran, 2021 | Mixed Method study, follow-up to RCT, RCT participants: quantitative data n = 170; quantitative data n = 14, Singapore | Habits-GDM | To follow-up on an mHealthf app-based lifestyle coaching program to measure the app’s usage behavior and to explore users’ perceptions of its usefulness in GDM management | Nutrition support  Educational materials  Customized database of Chinese, Malay, and Indian foods for logging | Messaging, glycemic control, nutrition support, and PA  Chat function with HCPs to answer patient questions within 24 hours; if a BGL was high patients were cued through automated messages to record their diet in the preceding 2 to 4 hours |
| Steinberg, 2021 | Pilot study (usability assessment), n = 22 low-income GDM or T2 pregnant patients, US | SweetMama | To examine patient characteristics associated with user engagement with a novel app for diabetes support during pregnancy | Nutrition support  Educational materials  Health literacy; focused on health literacy for the low-income demographic  App customized use of local food sources | Messaging and educational materials  Content was delivered tailored to gestational age, sent 3 or more times per week, with the ability to interact and obtain more health and nutrition information from a resource library; appointment reminders and motivational messaging |
| Yee, 2021 | Qualitative assessment with focus groups, n = 16 low-income GDM or T2 pregnant patients and n = 29 providers, US | SweetMama | To use focus groups to gain feedback from patients and HCPs on the visual appeal, architecture, function, and content of the app | Nutrition support  Educational materials  Health literacy  Customized content was delivered via simple straightforward language tailored for health literacy to a sixth-grade reading level | Messaging and educational materials  Individual goals were set by patients and HCPs during routine healthcare visits and delivered weekly via the app to serve as reminder and benchmark for goal-oriented behaviors; motivational messaging |
| Varnifield 2021 | Feasibility and acceptability study, n = 40 GDM patients, Australia | Mother | To evaluate the adoption and multidisciplinary care coordination of the app with first-time GDM patients | None | Live chat, messaging, glycemic control, nutrition support, PA,  and educational materials  Health messages were individualized to each patient’s needs through the clinician web portal |
| Seo, 2020 | Case Series Study, n = 4 GDM patients, Korea | mAPP | To evaluate the utility of a mobile app in nutritional intervention | None | Messaging, glycemic control, nutrition support, PA, medication support, educational materials, and postpartum care  The app collected clinical data (BGLs) and any messages sent to providers, nutritionist conducted nutrition assessment 2 times per month and delivered education to each patient; feedback regarding food choices, meal planning, with sample recipes |
| Garnweidner-Holme, 2020 | RCT, n = 238 GDM patients, Norway | Pregnant+ | To determine the impact culturally tailored dietary information and BGL monitoring on dietary behavior | Nutrition support  Educational materials  Language tailoring  Culturally tailored health and nutrition information offered in Norwegian, Urdu, and Somali | Messaging, glycemic control, nutrition support, PA, educational materials  Patients who logged high BGLs were referred to the app’s dietary recommendation page |
| Borgen, 2019 | RCT, n = 238 GDM patients, Norway | Pregnant+ | To assess the effect of the app on the 2-hour BGL of the routine postpartum  OGTTg | Nutrition support  Educational materials  Language tailoring  Culturally tailored health and nutrition information offered in Norwegian, Urdu, and Somali | Messaging, glycemic control, nutrition support, PA, educational materials, and postpartum care  Patients had the opportunity to write down personal goals and learn the advantages of PA during pregnancy; patients received specific information about during pregnancy and postpartum |
| Skar, 2017, 2018 | Semi-structured interviews as a follow-up to an RCT using Pregnant+ app, n = 17 participants with GDM, Norway | Pregnant+ | To explore the experiences of women with GDM with controlling their BGL and receiving health and nutrition information through the app | Nutrition support  Educational materials  Language tailoring  Culturally tailored health and nutrition information offered in Norwegian, Urdu, and Somali | Messaging, glycemic control, nutrition support, PA, educational materials, and postpartum care  Patient goal setting and materials on benefits of PA; received specific feedback during pregnancy and postpartum |
| Khalil, 2019 | Qualitative study with semi-structured interviews, n = 20 healthcare providers, n = 15 myDiabby users, France | myDiabby | To understand, from patients’ and HCPs’ perspectives about what drives the adoption and diffusion of a telemonitoring solution | None | Messaging, glycemic control, nutrition support, and medication support  HCPs reviewed patient BGLs and food logs to send alerts, adjust insulin dosage, and privately chat with patients with high BGL readings |
| Guo, 2019 | RCT, n = 124 GDM patients, China | Dnurse | To explore the effects of a mHealth intervention on pregnancy weight management, BGL control and pregnancy outcomes | None | Live chat, messaging, glycemic control, nutrition support, PA, medication support, and educational materials  Nurses reviewed patient BLGs and provided personalized dietary guidance; automated messages on underlying cause of abnormal BGLs were sent to patients when they logged an abnormal BGL reading |
| Miremberg, 2018 | RCT, n = 120 newly diagnosed GDM patients, US | GlucoseBuddy | To study the impact of a mobile daily feedback and communication between GDM patients and HCPs on patient compliance, glycemic control, pregnancy outcome and patient satisfaction | None | Live chat, messaging, glycemic control, nutrition support, PA, medication support, educational materials, and postpartum care  Daily communication and feedback system for patients and HCPs allowed ability to respond to patient concerns, reassure, or modify individual patient treatment plan on a daily basis |
| Mackillop, 2018 | RCT, n = 203 GDM patient, UK | GDm-Health | To determine the impact a mobile phone-based real-time blood glucose management system on controlling blood glucose levels | None | Messaging, glycemic control, nutrition support, medication support, and educational materials  Diabetes midwives reviewed patient BGLs 3 times a week and provided personalized feedback on diet, medication dose adjustments; automated alerts for high fasting BGLsh |
| Rigla, 2018 | Pilot Study, n = 20 patients diagnosed with GDM | MobiGuide | To test the feasibility and acceptance of a mobile decision support system for GDM; developed to include computer interpretable clinical practical guidelines, access to data from electronic health records (glucose, blood pressure, and activity sensors) | None | Messaging, glycemic control, PA  Patient-centered mobile decision support system for patients and HCPs using mobile sensors and self-reporting symptoms and receiving feedback through a mobile app |
| Peleg, 2017 | Feasibility, Quasi-Experimental, Spain, n = 19 Intervention GDM patients, n = 247 historical cohort GDM patients | MobiGuide | To assess the feasibility of Mobi Guide in two different groups of patients: 29 in GDM in Spain and 10 atrial fibrillation patients in Italy | None | Messaging, glycemic control, PA  Support system delivers continuous recommendations to the patient, shared decision-making embodies patient preferences, semantic date and integration; notification services |
| Smith, 2022 | Qualitative need and functional assessment using focus groups, n = 10 women with GDM, UK | Stay-Active | To describe the design and development of Stay-Active, using focus groups to explore barriers and facilitators to physical activity in women with GDM | None | Live chat, messaging, PA, educational materials  Patient goal setting and personalized feedback on performance and behavior goals; chat with HCPs over the phone or via text |
| Sung, 2019 | Prospective randomized controlled pilot study, n = 2, Korea | App #1 | To develop and evaluate a model for the management of GDM with the use of mobile health care | None | Messaging, glycemic control, nutrition support, PA, medication support, educational materials, and postpartum care  HCPs checked patient data and messages and send return messages with tailored medical and nutritional guidance; after delivery obstetric outcomes collected and OGTT performed |
| Jo, 2016 | Qualitative on-line survey to collect data to guide R&D, n = 5 participants recruited online, 2 were pregnant and 3 gave birth in the last 12 months, Korea | App #2 | To develop and evaluate an app that provided tailored recommendations based on lifestyle and clinical data entered by the user | None | Messaging, glycemic control, nutrition support, PA, and educational materials  Tailored recommendations from in-app algorithms based on patient data |
| Mohd Rosli, 2020 | Functional, n = 5 women with GDM, Malaysia | App #3 | To describe the design and development of a mobile app for GDM monitoring and to test the functionality of the app with women with GDM | None | Messaging, glycemic control, nutrition support  Patients received personalized meal recommendations based on their BGLs |
| Pais, 2021 | Qualitative need and feasibility assessment using semi-structured interviews, n = 13 healthcare providers with prior experience in providing care for women with GDM, n = 10 women with GDM, New Zealand | App #4 | To describe the user-centered design process used to develop a GDM self-management app, using semi-structured interviews with healthcare providers and women with GDM | None | Messaging, glycemic control, nutrition support, PA, medication support  Healthcare providers can view patient data (BGLs, food description and portion size, exercise, and weight) and write emails to their patients with personalized feedback |
