# Supplementary material for: Personalized and Culturally Tailored Features of Mobile Apps for Gestational Diabetes Mellitus and Their Impact on Patient Self-Management: Scoping Review
Source: JMIR Diabetes. 2024 Dec 12;9:e58327. doi: 10.2196/58327 (PMC11683422; doi:10.2196/58327)
Supplement: Multimedia Appendix 2 [file diabetes-v9-e58327-s002.docx]

**Multimedia Appendix 2.** Presentation of 3 mobile apps for GDM used in 7 studies including app name, study and study design, personalized and culturally tailored features, and the impact of GDM self-management.

| **App Name** | **Study (first author, and date)** | **Study Design, Participants, Country** | **Personalization in App Features in the Study** | **Cultural Tailoring in App Features in the Study** | **Impact on GDM Self-management** |
| --- | --- | --- | --- | --- | --- |
| Habits-GDM | Yew, 2021 | RCT^a^, n = 340 GDM^b^ patients, Singapore | Messaging, glycemic control, nutrition support, and PA^c^  Chat function with HCPs^d^ to answer patient questions within 24 hours; when a BGL^e^ was high patients were cued through automated messages to record their diet in the preceding 2 to 4 hours | *Nutrition support*  Customized database of Chinese, Malay, and Indian foods for logging | The app combined with standard care did not reduce excessive gestational weight gain among women with GDM^b^ but resulted in better maternal glycemic control and composite neonatal outcomes |
| Habits-GDM | Surendran, 2021 | Mixed Method study, follow-up to RCT, RCT participants: quantitative data n = 170; quantitative data n = 14, Singapore | Messaging, glycemic control, nutrition support, and PA  Chat function with HCPs to answer patient questions within 24 hours; if a BGL was high patients were cued through automated messages to record their diet in the preceding 2 to 4 hours | *Nutrition support*  Customized database of Chinese, Malay, and Indian foods for logging | The usage frequency varied greatly; the coaching and diet tracking features presented difficulties with food searching (food database was limited and not localized); negative aspects of the coaching were HCP’s^c^ lack of direct access to patient dashboards and logistical issues scheduling appointments |
| SweetMama | Steinberg, 2021 | Pilot study (usability assessment), n = 22 low-income GDM or T2 pregnant patients, US | Messaging and educational materials  Content was delivered tailored to gestational age, sent 3 or more times per week, with the ability to interact and obtain more health and nutrition information from a resource library; appointment reminders and motivational messaging | *Nutrition support*  *Educational materials*  *Health literacy*  App customized use of local food sources; focused on health literacy for the low-income demographic | Participants with GDM had on average greater minutes of use per session than participants with T2D^d^; participants with greater electronic health literacy, lower patient activation, and greater self-efficacy demonstrated greater total duration of use; no user metrics differed significantly by participant general health literacy |
| SweetMama | Yee, 2021 | Qualitative assessment with focus groups, n = 16 low-income GDM or T2D pregnant patients and n = 29 providers, US | Messaging and educational materials  Individual goals were set by patients and HCPs during routine healthcare visits and delivered weekly via the app to serve as reminder and benchmark for goal-oriented behaviors; motivational messaging | *Nutrition support*  *Educational materials*  *Health literacy*  Customized content was delivered via simple straightforward language tailored for health literacy to a sixth-grade reading level | Both patients and HCPs expressed satisfaction with the information and its presentation; participants positively reviewed the app’s goal-setting activity, which patients and HCPs felt helped focus patients’ efforts during pregnancy |
| Pregnant+ | Garnweidner-Holme, 2020 | RCT, n = 238 GDM patients, Norway | Messaging, glycemic control, nutrition support, PA, educational materials  Patients who logged high BGLs were referred to the app’s dietary recommendation page | *Nutrition support*  *Educational materials*  *Language tailoring*  Culturally tailored health and nutrition information offered in Norwegian, Urdu, and Somali | The app combined with usual care did not have any significant effect on the dietary behavior of the participants during pregnancy compared to the participants receiving usual care only; all participants using the app showed significant improvement in healthy eating behaviors compared to the baseline |
| Pregnant+ | Borgen, 2019 | RCT, n = 238 GDM patients, Norway | Messaging, glycemic control, nutrition support, PA, educational materials, and postpartum care  Patients had the opportunity to write down personal goals and learn the advantages of PA during pregnancy; patients received general information about maintaining a healthy lifestyle and specific information about their follow-up during pregnancy and postpartum | *Nutrition support*  *Educational materials*  *Language tailoring*  Culturally tailored health and nutrition information offered in Norwegian, Urdu, and Somali | The app combined with usual care did not have any significant effect on the 2-hour BGL^f^ of postpartum OGTT^g^ compared to the participants receiving usual care only |
| Pregnant+ | Skar, 2017, 2018 | Semi-structured interviews as a follow-up to an RCT using Pregnant+ app, n = 17 participants with GDM, Norway | Messaging, glycemic control, nutrition support, PA, educational materials, and postpartum care  Patient goal setting and materials on benefits of PA; received specific feedback during pregnancy and postpartum | *Nutrition support*  *Educational materials*  *Language tailoring*  Culturally tailored health and nutrition information offered in Norwegian, Urdu, and Somali | Users experienced an increase in their confidence in GDM self-management and increased motivation for behavior change; the information was considered easily accessible and reliable; technological challenges and lack of support from HCPs limited the use of the app for several patients |

^a^RCT: randomized controlled trial.

^b^GDM: gestational diabetes mellitus.

^c^PA: physical activity.

^d^HCP: health care provider.

^e^BGL: blood glucose level(s).

^f^mHealth: mobile health.

^g^OGTT: oral glucose tolerance test.

^h^Fasting BGL: a BGL taken in the morning upon waking before eating anything.
